# Brain serotonin 4 receptor binding is inversely associated with verbal memory recall

**DOI:** 10.1002/brb3.674

**Published:** 2017-03-17

**Authors:** Dea S. Stenbæk, Patrick M. Fisher, Brice Ozenne, Emil Andersen, Liv V. Hjordt, Brenda McMahon, Steen G. Hasselbalch, Vibe G. Frokjaer, Gitte M. Knudsen

**Affiliations:** ^1^ Neurobiology Research Unit and Center for Integrated Molecular Brain ImagingThe Neuroscience CentreRigshospitaletCopenhagenDenmark; ^2^ Department of BiostatisticsUniversity of CopenhagenCopenhagenDenmark; ^3^ Department of NeurologyThe Neuroscience CentreDanish Dementia Research CentreRigshospitalet, University of CopenhagenCopenhagenDenmark

**Keywords:** 5‐HT_4_ receptor, affective words, latent variable model, negative biases, positron emission tomography

## Abstract

**Background:**

We have previously identified an inverse relationship between cerebral serotonin 4 receptor (5‐HT_4_R) binding and nonaffective episodic memory in healthy individuals. Here, we investigate in a novel sample if the association is related to affective components of memory, by examining the association between cerebral 5‐HT_4_R binding and affective verbal memory recall.

**Methods:**

Twenty‐four healthy volunteers were scanned with the 5‐HT_4_R radioligand [^11^C]SB207145 and positron emission tomography, and were tested with the Verbal Affective Memory Test‐24. The association between 5‐HT_4_R binding and affective verbal memory was evaluated using a linear latent variable structural equation model.

**Results:**

We observed a significant inverse association across all regions between 5‐HT_4_R binding and affective verbal memory performances for positive (*p* = 5.5 × 10^−4^) and neutral (*p* = .004) word recall, and an inverse but nonsignificant association for negative (*p* = .07) word recall. Differences in the associations with 5‐HT_4_R binding between word categories (i.e., positive, negative, and neutral) did not reach statistical significance.

**Conclusion:**

Our findings replicate our previous observation of a negative association between 5‐HT_4_R binding and memory performance in an independent cohort and provide novel evidence linking 5‐HT_4_R binding, as a biomarker for synaptic 5‐HT levels, to the mnestic processing of positive and neutral word stimuli in healthy humans.

## 1 Introduction

Memory is vital for normal functioning in everyday life and accordingly memory problems are some of the most commonly reported symptoms in neurological and psychiatric disorders, such as major depressive disorder (MDD; Austin, Mitchell, & Goodwin, 2001; Gallassi, Di Sarro, Morreale, & Amore, 2006; Porter, Gallagher, Thompson, & Young, 2003). MDD patients in general experience not only memory impairments, but also present with specific negative affective memory biases. These memory biases are proposed to contribute to the emotional imbalance seen in MDD, by favoring negative information over positive information at different levels of information processing (Dalgleish, 2004; Elliott, Zahn, Deakin, & Anderson, 2011). As such, memory biases may sustain or worsen the depressed state or in the case of healthy individuals signify increased risk of developing MDD. Therefore, elucidating plausible molecular mechanisms that support memory and affective biases in the healthy brain is critical to advance our understanding of vulnerability to psychopathology such as MDD.

The serotonin (5‐HT) system is involved in memory processes in both humans and animals (Buhot, 1997; Meneses, 1999, 2013). Impairment of the 5‐HT system is also considered an important etiological factor in MDD (Buhot, Martin, & Segu, 2000; Krishnan & Nestler, 2008), where the 5‐HT system is the main target for antidepressant treatment (Morilak & Frazer, 2004). Thus, it is plausible that underlying molecular mechanisms may link affective verbal memory processes with risk and resilience architectures for brain disorders with affective symptomatology. This hypothesis is supported by outcomes of pharmacological and dietary manipulations of the 5‐HT system; it has consistently been shown that healthy volunteers who have low cerebral 5‐HT levels after undergoing acute tryptophan depletion exhibit worsened verbal memory consolidation as compared to baseline (Mendelsohn, Riedel, & Sambeth, 2009; Sambeth et al., 2009), and they have an impaired recall for positive and neutral words as compared to negative words (Kilkens, Honig, van Nieuwenhoven, Riedel, & Brummer, 2004; Klaassen, Riedel, Deutz, & Van Praag, 2002). Conversely, administration of selective serotonin reuptake inhibitors, which putatively increase central 5‐HT levels, is associated with enhanced positive affective memory processing (Harmer, 2008; Merens, Willem Van der Does, & Spinhoven, 2007).

Positron emission tomography (PET) shows that 5‐HT 4 receptors (5‐HT_4_R) are particularly abundant in brain regions involved in affective processing and memory, including the hippocampus, amygdala, and frontal cortex (Eglen, Wong, Dumuis, & Bockaert, 1995; Lucas, 2009). Our research group has recently published a high‐resolution in vivo atlas of the serotonin system in humans, including the 5‐HT_4_R (Beliveau & Ganz, 2017; downloadable maps are available at this website: https://nru.dk/FS5ht-atlas). Experimental studies also show that pharmacological stimulation of 5HT_4_R improves memory consolidation (Bockaert, Claeysen, Compan, & Dumuis, 2008; King, Marsden, & Fone, 2008), possibly through increased release of acetylcholine (Bockaert, Claeysen, Compan, & Dumuis, 2004). In addition, individuals resilient to develop MDD in spite of a familial predisposition have lower striatal 5‐HT_4_R binding (Madsen et al., 2014) and preclinical evidence suggests that the 5HT_4_R is a potential target for fast‐acting antidepressant treatment (Vidal et al., 2014). We recently provided novel evidence for a link between 5‐HT_4_R as imaged by PET and episodic memory performance in healthy humans, where hippocampal 5‐HT_4_R binding was found to be inversely related to recall of nonaffective words using the Rey Auditory Verbal Learning Task (RAVLT; Haahr et al., 2013). However, the association between 5‐HT_4_R binding and recall of affective words was not examined and ceiling effects on RAVLT performances motivated a follow‐up study in a novel cohort.

Here, we evaluate the association between 5‐HT_4_R binding and performance on the Danish Verbal Affective Memory Test‐24 (VAMT‐24) in a healthy population. In addition to probing the association between brain 5‐HT signaling and affective memory, this allowed us to evaluate if our previous findings could be replicated within a novel cohort. Based on our previous findings, we hypothesized to see a negative association between 5‐HT_4_R binding and recall of positive, negative, and neutral words. We further expected to find 5‐HT_4_R‐related differences in recall of positive, negative, and neutral words.

## 2 Methods and Materials

### 2.1 Participants

Twenty‐four healthy participants (three women) were recruited through Internet and newspaper advertisement. Eligible participants were screened for current and previous psychiatric symptoms, relevant medical history, alcohol, tobacco, illegal drug use, and abnormal blood tests. They also underwent a neurological examination by a trained clinician. Exclusion criteria for this study were significant medical history, which included psychiatric disorders, head trauma, a family history of psychiatric disorders, drug and alcohol abuse, and current or previous use of psychoactive drugs. Age ranged from 20 to 45 years (age = 26.7 ± 6.4, mean ± *SD*) and body mass index (BMI) ranged from 19 to 31 kg/m^2^ (BMI = 23.6 ± 3.0, mean ± *SD*; Table 1). Educational scores were rated on a 5‐point Likert scale; 1 (no vocational degree), 2 (<2 years of vocational education), 3 (2—4 years of vocational secondary education), 4 (2—4 years of academic education including a prior high school degree) to 5 (>4 years of academic education including a prior high school degree). Educational scores ranged from 1 to 5 (education = 4.0 ± 1.3, mean ± *SD*). Genotype information for the BDNF val66met and 5‐HTTLPR polymorphisms were available for all participants. Genotypes were determined as previously described (Madsen et al., 2016). Written informed consent was obtained and the study was registered and approved by the Copenhagen municipality (VEK [KF] 01‐2006‐20) and the Capital Region Ethics Committee (VEK H‐1‐2010‐085). Some of the included participants have previously been part of a publication relating BDNF val66met and 5‐HTTLPR polymorphisms and 5‐HT_4_R binding (Fisher, Holst, et al., 2015; Fisher et al., 2012), in methodology‐based papers (Greve et al., 2014; Haahr et al., 2014), and as healthy controls in a sample enriched with individuals with a family history of MDD (Madsen et al., 2014).

**Table 1:** Descriptive data

Measures (*n* = 24)	Mean ± *SD*	Minimum	Maximum
Age in years	26.7 ± 6.4	20	45
Body mass index (kg/m^2^)	23.6 ± 3.0	19	31
Sex (% male)	87.5		
Injected mass (μg)	1.4 ± 0.4	0.7	2.3
Education	4.0 ± 1.3	1	5
Frontal cortex BP_ND_	0.68 ± 0.08	0.49	0.81
Amygdala BP_ND_	0.89 ± 0.19	0.55	1.29
Hippocampus BP_ND_	1.04 ± 0.13	0.79	1.41
ACC cortex BP_ND_	0.82 ± 0.11	0.64	1.07
IMM Recall_Pos_	24 ± 5.2	13	34
STM Recall_Pos_	4.3 ± 1.8	0	8
LTM Recall_Pos_	4.6 ± 1.8	2	8
IMM Recall_Neg_	23.4 ± 5.0	14	33
STM Recall_Neg_	5.0 ± 1.7	2	8
LTM Recall_Neg_	5.0 ± 1.8	1	8
IMM Recall_Neu_	26.8 ± 5.1	18	37
STM Recall_Neu_	6.0 ± 1.4	3	8
LTM Recall_Neu_	5.9 ± 1.5	3	8

ACC cortex, anterior cingulate cortex; BP_ND_, binding potential; IMM, immediate memory recall; STM, short‐term memory recall; LTM, long‐term memory recall; Recall_Pos_, recall of positive words; Recall_Neg_, recall of negative words; Recall_Neu_, recall of neutral words.

### 2.2 Measures

#### 2.2.1 Verbal Affective Memory Task‐24

The VAMT‐24 is a newly validated 24‐word Danish affective memory test for use in healthy volunteers, developed by our research group (Jensen et al., 2016). It is a computerized test which includes three conditions: (1) *Learning and Immediate recall* (IMM), in which participants view 24 words on a computer screen (list A‐24) and are instructed to recall as many as possible. This procedure is repeated a total of five times (app. 15 min); (2) *Short‐term recall* (STM), in which participants view an interference list of 24 words on the computer screen (I‐24) and are instructed to recall list A‐24 (app. 5 min); and (3) *Long‐term recall* (LTM), in which participants are asked to do a surprise recall of list A‐24 after a period of 30 min (app. 5 min). Word lists display a fixed, counterbalanced order of words with respect to valence and contain eight positive (four adjectives, four nouns), eight negative (four adjectives, four nouns), and eight neutral (eight nouns) words. Each word trial displays a fixation cross (750 ms) and a word (750 ms) in black (font *= *times, size = 40) on a grey background. The screen (resolution = 1,680 × 1,050 pixels) is viewed from a distance of approximately 60 cm.

#### 2.2.2 PET and magnetic resonance imaging

[^11^C]SB207145 was synthesized as previously described (Haahr et al., 2014). Immediately after an intravenous bolus injection of [^11^C]SB207145, a 120 min dynamic 3D PET scan (6 × 5 s, 10 × 15 s, 4 × 30 s, 5 × 120 s, 5 × 300 s, and 8 × 600 s) was initiated using a high‐resolution research tomograph with an approximate in plane resolution of 1.5 mm. The scans were reconstructed using the iterative PSF reconstruction with attenuation map improvements (Hong et al., 2007; Sureau et al., 2008). Magnetic resonance imaging (MRI) was conducted on a 3T Siemens Magnetom Trio scanner (Erlangen, Germany). High‐resolution 3D sagittal magnetization prepared rapid gradient echo T1‐weighted sequences (TE/TR/TI = 3.04/1,550/800 ms, flip angle = 9°, in‐plane matrix = 256 × 256, in‐plane resolution = 1 × 1 mm, number of slices = 192, slice thickness = 1 mm, no gap) and high‐resolution 2D variable flip angle, sagittal turbo spin echo T2‐weighted sequences (TE/TR = 354/3,000 ms, 1 slab, slice resolution = 100%, bandwidth = 752 Hz/Pixel, echo spacing = 3.58 ms, turbo factor = 197, field of view = 282 mm, 1.1 × 1.1 × 1.1 mm voxels) were acquired and corrected for spatial distortions and nonuniformity. The T1‐ and T2‐weighted MRIs were used to segment the brain into gray and white matter and cerebrospinal fluid using VBM5 (Wellcome Department of Cognitive Neurology, London, UK) and each voxel was assigned to the tissue class with the highest probability and this segmentation was subsequently used for delineation of the region of interest. The T2‐weighted images served for brain masking purposes. To determine single‐subject within PET scan motion and realignment, the automatic image registration algorithm was used (Woods, Cherry, & Mazziotta, 1992). PET scans were smoothened using a 10 mm within‐frame Gaussian filter before alignment. We estimated rigid translation/rotation parameters aligning each PET frame to a single PET frame with sufficient structural information using the scaled least squares cost‐function (frame 26: 20–25 mins postinjection). Co‐registration of high‐resolution MR and PET images was performed using SPM8 based on the mean of frames 10–26, corresponding to a flow‐weighted image. Accurate co‐registration was confirmed by visual inspection across all planes.

Pvelab was used to automatically delineate regions from the participant's structural MRI scan and time‐activity curves within each region were determined (Svarer et al., 2005). The binding potential (BP_ND_) of [^11^C]SB207145 was modeled with the simplified reference tissue model using PMOD (PMOD, Zurich, Switzerland) with cerebellum as a reference region (Marner et al., 2009), defined as: BP_ND_ = *f*_ND_ × *B*_avail_ × (1/*K*_D_), where *f*_ND_ is the free fraction of ligand in the nondisplaceable tissue compartment, *B*_avail_ is the concentration of receptors available for binding, and *K*_D_ is the dissociation constant (Innis et al., 2007). In total, four regions were included in our model: frontal cortex, amygdala, hippocampus, and anterior cingulate cortex as these regions are commonly associated with memory and affect regulation (Eglen et al., 1995; Elliott, Rubinsztein, Sahakian, & Dolan, 2002). The frontal cortex region was delineated as a volume‐weighted sum of orbitofrontal cortex, medial inferior frontal gyrus, and superior frontal gyrus, which were defined from the parcellation results using Pvelab.

#### 2.2.3 Statistics

Statistical analyses were carried out in SPSS (v20.0) and R (v3.0.2; R Core Team, 2013). The lava package in R was used to obtain maximum likelihood estimates. IMM scores were divided by five in order to obtain an equivalent scale to STM and LTM scores. We examined the association between positive, negative, and neutral word recall and 5‐HT_4_R binding using a linear latent variable model; a flexible structural equation model that allows for explicitly testing global and specific effects within a single model. Latent variable models can be seen as an extension of linear mixed models for the analysis of multiple repeated measurements (e.g., BP_ND_ and memory). While linear mixed models parameterized with a random slope assume a constant correlation between measurements, latent variable models have the additional benefit of relaxing this assumption allowing certain measurements to be more correlated than others (i.e., measurements from certain pairs of regions can be more correlated compared with measurements from other regions.

**Figure 1:**
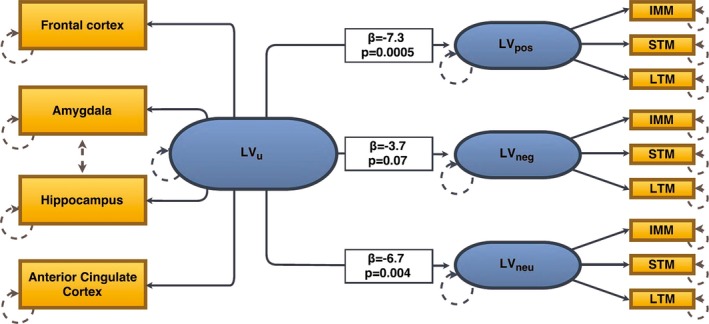
A schematic overview of the latent variable structural equation model. For clarity, covariates are not delineated in the model. The blue ovals represent the four latent variables (LV_pos_, LV_neg_, and LV_neu_ = memory component and LV_u_ = 5‐HT_4_R component). The orange boxes predicted by the latent variable LV_u_ represent measured regional 5‐HT_4_R binding potential values. The orange boxes predicted by the latent variables LV_pos_, LV_neg_, and LV_neu_ represent measured immediate (IMM), short term (STM), and long‐term (LTM) memory recall for the respective word category (i.e., positive, negative, and neutral). The hatched gray line between hippocampus and amygdala indicates additional shared correlation and the hatched gray circles indicate they are estimated with error. Parameter estimates (β) and *p*‐values are noted for the three paths between LV_u_ and LV_pos_, LV_neg_, and LV_neu_

**Figure 2:**
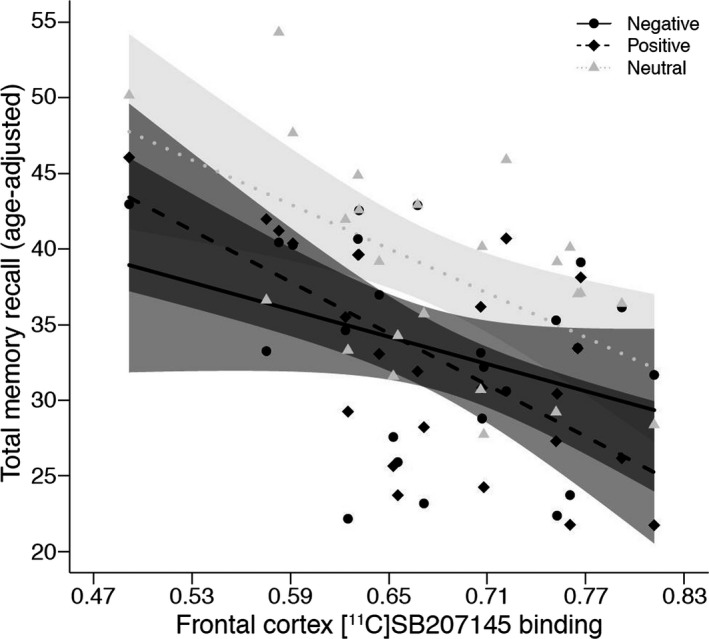
A grouped scatter plot of 5‐HT_4_R binding potential values plotted against total words recalled (i.e., sum of immediate, short term, and long‐term recall of positive, negative, and neutral words). Lines and shading for each line represent slope estimates and 95% confidence intervals, respectively. Data shown are adjusted for age. Our data indicate an inverse relation between memory recall and 5‐HT_4_R binding. They also suggest a steeper slope for positive and neutral word recall compared to negative and that the relative recall between positive and negative words changes with 5‐HT_4_R binding such that more negative words relative to positive words are recalled with increasing binding and the opposite with decreasing binding

Consistent with a previous study from our research group (Fisher, Holst, et al., 2015), our model included the shared correlation between regional binding in frontal cortex, amygdala, hippocampus, and anterior cingulate gyrus modeled as one latent variable (LV_u_). IMM, STM, and LTM recall scores were highly inter‐correlated for each word category (all *r* > .61). Hence, the shared correlation between IMM, STM, and LTM recall scores for each word category (i.e., positive, negative, and neutral word recall) was modeled as three separate memory latent variables (positive, LV_pos_, negative, LV_neg_, neutral, LV_neu_). We modeled a correlation between LV_u_ and LV_pos_, LV_neg,_ and LV_neu,_ reflecting the association between 5‐HT_4_R binding and memory performance (Figure 1). A likelihood ratio test between this model and an identical model wherein the relations between LV_u_ and LV_pos_, LV_neg,_ and LV_neu_ were equivalent was used to evaluate differences across word categories in the associations with 5‐HT_4_R binding. An approximate visualization of the associations was made by plotting the sum scores (IMM, STM, and LTM word recall) for each word category against 5‐HT_4_R binding in the frontal cortex, which was used as reference scale in the model (Figure 2). Model fit was evaluated by comparing the model to a saturated model using a likelihood ratio test.

Age was included as a covariate of all latent variables given previous evidence supporting the effects on 5‐HT_4_R binding and memory (Grady & Craik, 2000; Madsen, Haahr, et al., 2011). Consistent with our previous observations (Fisher, Holst, et al., 2015; Fisher et al., 2012), a region‐specific effect of 5‐HTTLPR on frontal cortex binding was included. Sex was omitted as a covariate because there were so few women in the sample. BMI, education, BDNF val66met status, and [^11^C]SB207145 injected mass were considered but excluded from the final model because they did not statistically significantly predict binding or memory. Additional model paths were considered iteratively, based on Score tests of improvement in model fit with a false‐discovery rate of *q* < 0.05 across all possible paths. This test supported modeling additional covariance between the amygdala and hippocampus (*q* = 0.03), which was included in the model. No additional model paths were supported (*q* > 0.21), indicating that there was no substantial amount of residual shared correlation after modeling the association between latent variables.

## 3 Results

### 3.1 Descriptive data

Descriptive data are shown in Table 1. Most of the participants were males and the cohort was young. For 20 of the participants, an average of 7 days (range: 0–21 days) elapsed between VAMT‐24 assessment and the PET scan; the remaining 4 participants were tested within 7 months after the PET scans were acquired (average = 177 days, range: 163–211 days). The long‐term (app. 6 months) retest reliability of VAMT‐24 has been established (IMM, ICC = 0.84, STM, ICC = 0.77, LTM, ICC = 0.78; Jensen et al., 2016), and we therefore analyzed them as a single group. When we excluded the four participants from our analyses, we obtained similar results. All participants received an injected mass between 0.7 and 2.3 μg, which is well below 4.5 μg corresponding to less than 5% occupancy (Madsen, Marner, Haahr, Gillings, & Knudsen, 2011).

### 3.2 5‐HT_4_R binding and VAMT‐24 performance

Our data supported a latent variable model structure as indicated by high correlation in 5‐HT_4_R binding across regions (LV_u_: all factor loadings, *p* < 2.7 × 10^−4^) and a high correlation in memory performance across IMM, STM, and LTM scores for each word category (LV_pos_: all factor loadings, *p* < 8.3 × 10^−5^, LV_neg_: all factor loadings, *p* < 3.7 × 10^−7^, LV_neu_: all factor loadings, *p* < 2.1 × 10^−5^). Overall model fit was good. The applied model is shown in Figure 1.

Within our model, 5‐HT_4_R binding was negatively associated with positive word recall (Estimate, −7.3 [−11.4 to 3.2], *p* = 5.5 × 10^−4^; units: IMM positive words recalled per unit frontal cortical BP_ND_) and with neutral word recall (Estimate, −6.7 [−11.4 to −2.1], *p* = .004; units: IMM neutral words recalled per unit frontal cortical BP_ND_), but not with negative word recall (Estimate, −3.7 [−7.7 to 0.32], *p* = .07; units: IMM negative words recalled per unit frontal cortical BP_ND_). As expected, 5‐HTTLPR S‐carriers had lower 5‐HT_4_R binding than L_A_L_A_ carriers (*p* = .015). Age was significantly negatively associated with memory performance for each word category (all *p* < .024), but not with 5‐HT_4_R binding (*p* = .22). For results from all delineated model paths, please see supplementary material.

The likelihood ratio test to determine if there was a significant difference between the word category latent variables and LV_u_ was not significant (χ^2^ = 1.7, *df* = 2, *p* = .43). Thus, although the associations between 5‐HT_4_R binding and recall for positive and neutral words had steeper negative slopes than negative words (Figure 2), this difference did not reach statistical significance.

## 4 Discussion

In healthy volunteers, there is a significant inverse relationship between memory performance and 5‐HT_4_R binding for positive and neutral word recall but not for negative word recall. These findings confirm our previous findings of a negative association between 5‐HT_4_R binding and memory performance (Haahr et al., 2013), in a novel sample using a test with no ceiling effects and which includes an affective component. The observed associations between in vivo 5‐HT_4_R binding and memory for the included regions appeared to be regulated in a global manner and across memory processes, that is encoding, retrieval, and consolidation. Overall, these findings reinforce a link between an endogenous feature of brain 5‐HT signaling and memory performance; a critical and often lacking piece of evidence supporting observed brain‐behavior relations traditionally studied by exogenous dietary or pharmacological manipulations of the 5‐HT system.

In animals, converging evidence supports a potent role for 5‐HT_4_R in memory and learning (Bockaert et al., 2008; Meneses, 2013), and attenuated response to stress and novelty with 5‐HT_4_R agonism (Compan et al., 2004). Systemic injections of 5‐HT_4_R agonists or partial agonists improve performance in a broad spectrum of memory tasks (Bockaert et al., 2004; Marchetti, Dumuis, Bockaert, Soumireu‐Mourat, & Roman, 2000), however, not always in a straight forward manner as 5‐HT_4_R agonists impaired memory in young rats but improved it in old rats (Lamirault & Simon, 2001). Thus, our results do not directly reconcile with these animal models, where promnestic effects are generally seen with 5‐HT_4_R agonism (Manuel‐Apolinar et al., 2005). We suggest that extrapolating from animal studies that have investigated direct effects of 5‐HT_4_R agonism may not translate directly to the present findings, where the BP reflects a composite measure of both receptor density and affinity (Innis et al., 2007). However, experimental stimulation of the 5‐HT_4_R in rodents may reveal an important direct role of this receptor in specific memory processes, which remains to be clarified in humans.

There is some evidence that 5‐HT_4_R availability is inversely related to synaptic cerebral 5‐HT concentration; for example, 3 weeks of fluoxetine administration to healthy volunteers decreases 5‐HT_4_R binding (Haahr et al., 2014), as is the case for rats exposed to 3 weeks of paroxetine administration (Licht et al., 2009). From such a possible central 5‐HT tonus perspective, the observed inverse relationship between 5‐HT_4_R binding and memory in this study is, thus, consistent with the notion of high central 5‐HT tonus (i.e., lower 5‐HT_4_R binding) being coupled to better memory performance (Meneses, 1999). Thus, further studies are needed to elucidate the functional significance of 5‐HT_4_R binding and affective memory; preferably in a prospective set‐up with interventions either targeting (1) central 5‐HT tonus to see whether changes in 5‐HT_4_R availability correlate with changes in affective memory biases to specify its functional role in healthy individuals or (2) direct pharmacological stimulation of the 5‐HT_4_R and affective memory correlates.

Importantly, we here replicate our previous findings of an inverse association between memory performance and 5‐HT_4_R binding in healthy volunteers (Haahr et al., 2013) within an independent sample and using only high resolution PET scans. Also, the previously used memory paradigm, a 15‐word verbal learning task (RAVLT), exhibited severe ceiling effects, while the VAMT‐24 was specifically developed for use in healthy individuals and is less prone to ceiling effects compared to the RAVLT (Jensen et al., 2016). Thus, the current findings validate our previous study using a more sensitive memory test and importantly extend this finding with the use of an affective component of memory in relation to 5‐HT_4_R binding.

Of particular interest to the study of affective memory biases, we tested whether word categories (positive, negative, and neutral words) differed in their association with 5‐HT_4_R binding. The estimated association with 5‐HT_4_R binding for negative word recall was 49% lower compared to positive word recall and 44% lower compared to neutral word recall, however, these differences did not reach statistical significance. Such large differences in a sample limited in size (*n* = 24) suggest that statistical power may not have been sufficient to detect a significant difference between word categories. Thus, further studies with larger sample sizes are needed to confirm or reject the hypothesis of affective bias in the association between verbal memory performance and 5‐HT_4_R binding.

Many previous studies have demonstrated changes in affective processing in response to pharmacological manipulation of 5‐HT signaling (Fisher, Haahr, et al., 2015; Harmer, Bhagwagar, Cowen, & Goodwin, 2002; Harmer, Shelley, Cowen, & Goodwin, 2004; Mendelsohn et al., 2009; Molodtsova, 2008). Therefore, the noted differences in recall of negative words compared to positive and neutral words should also be investigated targeting the 5‐HT_4_R with 5‐HT acting interventions. Converging evidence supports that antidepressants modulate affective memory systems to ameliorate negative biases in depressive patients (Harmer, 2008; Harmer et al., 2009). The typical antidepressant response interval of 2‐3 weeks with SSRI compounds, during which there may be a restructuring of affective orientation, is consistent with the time interval over which antidepressants were shown to affect central 5‐HT_4_R in animal models and humans (Haahr et al., 2014; Licht et al., 2009; Marner et al., 2010). Thus, 5‐HT_4_R‐related effects could help explain the time course of antidepressants. Furthermore, future studies integrating a pharmacological challenge of the 5‐HT system with a PET radioligand sensitive to acute changes in brain 5‐HT levels would be warranted to elucidate how region‐specific dynamics are related to affective verbal memory performance.

### 4.1 Limitations

Although our findings provide evidence for an association between central 5‐HT_4_R binding and affective verbal memory recall, some limitations should be considered. Our sample size is small, which may have undermined our ability to disentangle effects on specific memory processes (i.e., encoding, consolidation, and retrieval) and word category specific effects. Notably, our findings do not appear to be driven by individual data points and the replication of a previously reported association between 5‐HT_4_R binding and memory performance in healthy volunteers supports the findings (Haahr et al., 2013). Also, due to the sex distribution (3 women), we were not able to address potential moderating effects of sex on the association between 5‐HT_4_R binding and affective memory. This is relevant to consider in future studies given the prevalence of MDD in women and reported sex differences in 5‐HT_4_R binding and other features of the 5‐HT system (Madsen, Haahr, et al., 2011; Moses‐Kolko et al., 2011).

## Conflict of Interest

GMK has received honoraria as Field Editor of the International Journal of Neuropsychopharmacology and as scientific advisor for H. Lundbeck A/S. VGF has received honorarium as speaker for H. Lundbeck A/S. All other authors declare that they have no conflicts of interest.
