# Supplementary material for: Brain serotonin 4 receptor binding is inversely associated with verbal memory recall
Source: Brain Behav. 2017 Mar 17;7(4):e00674. doi: 10.1002/brb3.674 (PMC5390847; doi:10.1002/brb3.674)
Supplement: Supplementary file 1 [file BRB3-7-e00674-s001.docx]

| **Supplementary table: Parameter and significance estimates for all model paths in the latent variable model** | | | | |
| --- | --- | --- | --- | --- |
| **Model paths** | **Estimate** | **SE** | **P-value** |  |
| LV_u_ -> Frontal cortex | 1 (fixed) |  |  |  |
| LV_u_ -> Amygdala | 1.5 | 0.4 | <0.001 |  |
| LV_u_ -> Hippocampus | 1.1 | 0.3 | <0.001 |  |
| LV_u_ -> ACC cortex | 1.4 | 0.2 | <0.0001 |  |
| LV_Pos_ -> IMM Recall_Pos_ | 1 (fixed) |  |  |  |
| LV_Pos_ -> STM Recall_Pos_ | 2.1 | 0.5 | <0.0001 |  |
| LV_Pos_ -> LTM Recall_Pos_ | 2.3 | 0.5 | <0.0001 |  |
| LV_Neg_ -> IMM Recall_Neg_ | 1 (fixed) |  |  |  |
| LV_Neg_ -> STM Recall_Neg_ | 1.7 | 0.3 | <0.0001 |  |
| LV_Neg_ -> LTM Recall_Neg_ | 2.1 | 0.4 | <0.0001 |  |
| LV_Neu_ -> IMM Recall_Neu_ | 1 (fixed) |  |  |  |
| LV_Neu_ -> STM Recall_Neu_ | 1.3 | 0.3 | <0.0001 |  |
| LV_Neu_ -> LTM Recall_Neu_ | 1.5 | 0.3 | <0.0001 |  |
| 5-HTTLPR -> Frontal cortex | -0.03 | 0.01 | 0.02 |  |
| LV_u_ -> Age | -0.003 | 0.002 | 0.2 |  |
| LV_u_ -> LV_Pos_ | -7.3 | 2.1 | 0.0005 |  |
| LV_Pos_ -> Age | -0.07 | 0.02 | 0.001 |  |
| LV_u_ -> LV_Neg_ | -3.7 | 2 | 0.07 |  |
| LV_Neg_ -> Age | -0.08 | 0.02 | 0.001 |  |
| LV_u_ -> LV_Neu_ | -6.7 | 2.4 | 0.004 |  |
| LV_Neu_ -> Age | -0.06 | 0.02 | 0.02 |  |
| **Note:** LV_u_: Latent variable of 5-HT_4_ binding, LV_Pos_: Latent variable of recall of positive words, LV_Neg_: Latent variable of recall of negative words, LV_Neu_: Latent variable of recall of neutral words, ACC cortex: Anterior cingulate cortex, BP_ND_: Binding potential, 5-HTTLPR: Serotonin transporter polymorphism, IMM: Immediate memory recall, STM: Short term memory recall, LTM: Long term memory recall, Recall_Pos_: recall of positive words, Recall_Neg_: recall of negative words, Recall_Neu_: recall of neutral words, SE: standard error. | | | | |
